# Book Reviews

**Published:** 1895-09

**Authors:** 


### Book Reviews, Etc.

Prof. Thomson's "Manual of Dietetics" is a work of great value, as a succinct exposition of the subject and a reliable guide. It is surprising that so little attention is paid this very important subject in systematic works on practice, when it is perfectly plain that diet is one of the weightiest factors in the treatment of the sick. This deficiency of detail has been admirably supplied in the "Manual of Dietetics" and the practitioner may find the appropriate diet for each disease which is influenced by right feeding.

The subject matter is divided into food and food preparations; stimulants, beverages and condiments; cooking, food preparation and preservation. The quantity of food required, food required for special conditions, food digestion; the general relation of food to special diseases; administration of food for the sick; diet in disease; diet in diseases of special organs, as urinary, alimentary, nervous systems, etc.; diet in miscellaneous diseases; rations and dietaries.

The book contains 802 pages, and is published by D. Appleton & Co., N. Y.

THE POCKET MATERIA MEDICA AND THERAPEUTICS.—A Resume of the Action and Doses of all Officinal and Non-Officinal Drugs Now in Common Use. By C. Henri Leonard, A.M., M. D., Professor of the Medical and Surgical Diseases of Women and Clinical Gynæcology in the Detroit College of Medicine; Member of the American Medical Association, etc., etc. Second Edition, revised and enlarged; cloth, large 16mo, 367 pages; price, post-paid, $1.00. Detroit, 1895: The Illustrated Medical Journal Co., Publishers.

The second edition of this handy little work has had 67 pages added besides typographical errors corrected.

The drugs are arranged alphabetically, synonym in English, French, and German, dose, habitat, botanical source, part used, if a plant; if a mineral, its physical characters. A cross index renders the quick finding of a drug very easy.

It can be well recommended to physician, druggist or student. The price is very moderate, $1.00.

HAYEM & HARE'S PHYSICAL AND NATURAL THERAPEUTICS—Physical and Natural Therapeutics; The Remedial Use of Heat, Electricity, Modifications of Atmospheric Pressure, Climates and Mineral Waters. By Georges Hayem, M. D., Professor of Clinical Medicine in the Faculty of Medicine of Paris. Edited with the assent of the author, by Hobart Amory Hare, M. D., Professor of Therapeutics in the Jefferson Medical College of Philadelphia. In one handsome octavo volume of 414 pages, with 113 engravings. Cloth, $3.00. Philadelphia: Lea Brothers & Co., Publishers, 1895.

The object of this book is to set forth in a precise form all information needful to the physician concerning health resorts, mineral waters, and method of treatment by the application of heat and cold, baths and electricity. The American editor has added chapters on American climate and mineral springs, and has rendered the text applicable to the American physician.

The work deserves high praise and should find its way into the library of every practitioner who wishes to be familiar with this important subject and the natural therapeutic resources of his own country.

